# Editorial for the Special Issue on Piezoelectric MEMS

**DOI:** 10.3390/mi9050237

**Published:** 2018-05-15

**Authors:** Ulrich Schmid, Michael Schneider

**Affiliations:** Institute of Sensor and Actuator Systems, TU Wien, 1040 Vienna, Austria

Electromechanical transducers that utilize the piezoelectric effect have been increasingly used in micro-electromechanical systems (MEMS) either as substrates or as thin films. Piezoelectric transducers feature a linear voltage response, no snap-in behaviour, and can provide both attractive and repulsive forces. Such features remove the inherent physical limitations present in the commonly used electrostatic transducer approach while preserving its beneficial properties such as low-power operation. Furthermore, piezoelectric materials are suitable for both actuation and sensing purposes; in addition to their compact design, they enable pure electrical excitation as well as read-out of the transducer element. On the basis of these characteristics, the operation of piezoelectric transducers suits a large variety of different application scenarios, ranging from resonators in advanced acoustic devices in liquid environments to sensors in harsh environments. To uncover the full potential of piezoelectric MEMS, interdisciplinary research efforts in a variety of subjects are needed, including investigations of advanced piezoelectric materials with regard to the design of novel piezoelectric MEMS sensor and actuator devices as well as the integration of PiezoMEMS devices into full low-power systems.

This special issue covers contributions to the current state of this exciting field of research in the following topics:

1. Experimental and theoretical research on the deposition, properties, and actuation structures of piezoelectric materials such as aluminum nitride (AlN) and lead zirconate titanate (PZT) with a focus on the application in MEMS devices. An et al. [1] presented an explanation of the causes of hysteresis effects in piezoelectric ceramic actuators using micropolarization theory. By employing a control method based on a tripartite Prandtl-Ishlinskii (PI) model, An et al. could improve the tracking performance by more than 80%. In addition, Qin et al. proposed a modification of the PI model which allows for the identification of all parameters of the hysteresis model through one set of experimental data, without the need for additional curve fitting [2]. Sette et al. [3] developed fully transparent PZT thin film capacitors contacted via Al-doped zinc oxide (AZO), which can be utilized to add new functionalities to transparent surfaces, such as providing in-display actuation for haptic feedback in mobile devices.
2. Modelling and simulation of piezoelectric MEMS devices and systems. Yang et al. [4] proposed and simulated a novel AlN on silicon cantilever gyroscope based on inversely connected electrode stripes, which offers a theoretical sensitivity of 0.145 pm/°/s at a small device footprint. Wei et al. established [5] general analytical equations based on a kinematic analysis of compliant bridge mechanisms, which were then used to optimize a piezo-driven compliant bridge mechanism. Li et al. [6] presented the dynamic characteristics of piezoelectric micro jets by utilizing a direct coupling simulation approach, including the impact of inlet and viscous losses. In a second paper, Li et al. [7] analyzed the impact of fluid density and acoustic velocity on the micro jet performance. Chen et al. [8] simulated and experimentally validated a PZT-actuated, triple-finger gripper, which reached an output resolution of 145 nm/V at a maximum displacement range of 43.4 µm.
3. Piezoelectric MEMS resonators for measuring physical quantities such as mass, acceleration, yaw rate, as well as the pressure, viscosity, or density of liquids. Pfusterschmied et al. [9] demonstrated that piezoelectric MEMS resonators with high quality factors in liquids can be used to monitor the change in grape must during wine fermentation, which is a direct quality indicator of the fermentation process. Yu et al. [10] presented a unique take on the MEMS gyroscope through use of the acoustic Sagnac effect, which measured the phase difference between two sound waves traveling in opposite directions in a circular MEMS structure actuated by PMUTs.
4. Acoustic devices, such as surface acoustic wave (SAW), bulk acoustic wave (BAW), or thin film bulk acoustic resonators (FBARs) as well as acoustic transducers, which use piezoelectric MEMS, such as microphones or loudspeakers. Udvardi et al. [11] proposed a low-volume, piezoMEMS-based spirally shaped acoustic receptor array. The device is small enough to be used in cochlear implants while maintaining a good low-frequency response with output voltages high enough for direct analog conversion. Mansoor et al. [12] presented a transduction system for extremely fast system dynamics, which can create stationary as well as traveling surface waves in a turbulent boundary layer.
5. Piezoelectric energy harvesting technologies. Xu et al. [13] presented a hybrid meso-scale energy harvesting device, which combines both piezoelectric and electromagnetic harvesting schemes; under certain conditions, this hybrid approach can provide wider bandwidths and higher output power for vibrational energy harvesters.
